# Supplementary material for: When and how particles are removed by drops
Source: Sci Adv. 2026 Nov 13;12(46):eaef8434. doi: 10.1126/sciadv.aef8434 (PMC13763941; doi:10.1126/sciadv.aef8434)
Supplement: Supplementary file 1 [file sciadv.aef8434_sm.pdf]

Science Advances
AAAS

Supplementary Materials for

When and how particles are removed by drops

Abhinav Naga *et al.*

Corresponding author: Abhinav Naga, abhinav.naga@ed.ac.uk; Doris Vollmer, vollmerd@mpip-mainz.mpg.de; Halim Kusumaatmaja, halim.kusumaatmaja@ed.ac.uk

Sci. Adv. **12**, eaef8434 (2026)
DOI: 10.1126/sciadv.aef8434

The PDF file includes:

Sections S1 to S4
Tables S1 to S3
Figs. S1 to S7
Legends for movies S1 to S7

Other Supplementary Material for this manuscript includes the following:

Movies S1 to S7

S1. DESCRIPTION OF SUPPLEMENTARY MOVIES

1. Movie 1: Experiment showing a water drop interacting with a hydrophilic and a hydrophobic particle on a hydrophobic surface. The interaction is imaged using the inverted laser scanning confocal microscope setup shown in Fig. 1C. The water drop is shown in gray. The particle appears black but its position can be deduced from the drop deformation.

2. Movie 2: Simulation showing a water drop interacting with a hydrophilic particle ($\theta_p = 30°$) on a hydrophobic surface ($\theta_s = 90°$).

3. Movie 3: Simulation showing a water drop interacting with a hydrophobic particle ($\theta_p = 90°$) on a hydrophobic surface ($\theta_s = 90°$).

4. Movie 4: Simulation showing a water drop interacting with a hydrophobic particle ($\theta_p = 100°$) on a superhydrophobic surface ($\theta_s = 150°$).

5. Movie 5: Simulation showing a water drop interacting with a hydrophobic particle ($\theta_p = 90°$) on a hydrophobic surface ($\theta_s = 90°$). Compared to all the other simulation movies where μ was set to 0.6 and 0.3 for the sliding and rolling friction, respectively, here the friction coefficient was higher ($\mu = 1.0$).

6. Movie 6: Simulation showing a water drop interacting with a superhydrophobic particle ($\theta_p = 150°$) on a hydrophobic surface ($\theta_s = 90°$).

7. Movie 7: Simulation showing a water drop interacting with a hydrophilic particle ($\theta_p = 30°$) on a hydrophilic surface ($\theta_s = 30°$).

S2. EFFECT OF CANTILEVER

To check whether the presence of the cantilever influenced the collision outcome, we repeated experiments by changing the position of the cantilever while keeping everything the same (Supplementary Figure S1). Changing the position of the cantilever causes different parts of the drop to deform, thus allowing us to check whether the drop deformation caused by the cantilever influences the collision outcome. In the first experiment, we positioned the cantilever at the rear of the drop to push the drop (Supplementary Figure S1a). In the second experiment, we positioned the cantilever at the front of the drop to pull the drop (Supplementary Figure S1b). The particle followed the same trajectory in both cases, resulting in the same final outcome.

Furthermore, we verified that the spacing Δz between the particle and the bottom edge of the cantilever also does not influence the collision outcome as long as Δz exceeds the particle diameter. In particular, in the example shown in Supplementary Figure S1, the particle moved around the drop perimeter and remained attached to the rear as Δz was increased from 300 μm to 500 μm. The same outcome was also observed when doubling the drop volume from 2 μL to 4 μL, highlighting that the outcome is also independent of the drop volume. For sufficiently large drops, we do not expect the drop volume to influence the outcome because the main forces that determine the outcome are the capillary force and the particle friction, both of which are independent of drop volume.

S3. EFFECT OF ROLLING

When rolling is the predominant mode of motion, the particle is expected to remain attached to the receding drop-air interface when

$$F_\gamma^x > F_r + F_{\Delta\theta_p}, \tag{S1}$$

where F_γ^x is the component of the capillary force along the direction of motion x, F_r is the rolling friction, and $F_{\Delta\theta_p}$ is the resistive force due to capillary torque. Rolling friction accounts for energy loss when the particle rolls on the surface. Capillary torque arises from contact angle hysteresis when the particle rotates at the drop-air interface.

Rolling friction can be described using (36)

$$F_r = \mu_r(F_p - F_\gamma^z), \tag{S2}$$

where μ_r is the coefficient of rolling friction, F_p is the downward contact force (*i.e.*, gravitational force in our simulations) between the particle and the surface, and F_γ^z is the component of the capillary force due to the drop in the vertical (upward) direction.

Combining Eqs. S1–S2, gives the following criterion for the successful capture of a rolling particle:

$$F_\gamma^x > \mu_r(F_p - F_\gamma^z) + F_{\Delta\theta_p}. \tag{S3}$$

By grouping all terms into a single dimensionless number, $(F_\gamma^x + \mu_r F_\gamma^z - F_{\Delta\theta_p})/(\mu_r F_p)$, and taking the maximum, we obtain the capillary capture parameter corresponding to a rolling particle:

$$C \approx \frac{\max(F_\gamma^x + \mu_r F_\gamma^z) - F_{\Delta\theta_p}}{\mu_r F_p}. \tag{S4}$$

The resistive force contribution due to capillary torque $F_{\Delta\theta_p}$ depends on the contact angle hysteresis of the particle and the precise geometry of the liquid–air interface around the particle. It was proposed that this contribution can be estimated as (44)

$$F_{\Delta\theta_p} \approx 4\gamma k_1 R_p \sin^2\theta_p \sin\Delta\theta_p, \tag{S5}$$

where $k_1 \approx 24/\pi^3$, θ_p is the equilibrium particle contact angle, and $\Delta\theta_p$ is the difference between the particle advancing and receding contact angles.

To determine whether sliding or rolling is the predominant mode of motion, the slip ratio $\omega R_p/V_p$, where ω is the angular velocity of the particle and V_p is its center of mass velocity, can be used. A ratio close to 1 indicates pure rolling, whereas a ratio close to 0 indicates pure sliding. In the phase diagram presented in Figure 4A of the manuscript, $\omega R_p/V_p$ is typically small and less than 0.5 for most cases. Therefore, sliding was the predominant mode of motion in these simulations.

S4. VAN DER WAALS FORCES BETWEEN GLASS AND PDMS

Van der Waals forces between glass and PDMS across water are attractive and therefore cannot explain the presence of a water film below a glass particle (Figure 5 in the main manuscript). The van der Waals forces are characterized by the Hamaker constant between the particle and the surface in water. The Hamaker constant between materials 1 and 2 separated by a thin layer of material 3 can be estimated by using the following equation from Lifshitz theory (36):

$$A_H = \frac{3}{4}k_B T\left(\frac{\epsilon_1-\epsilon_3}{\epsilon_1+\epsilon_3}\right)\left(\frac{\epsilon_2-\epsilon_3}{\epsilon_2+\epsilon_3}\right) + \frac{3h\nu_e}{8\sqrt{2}}\frac{(n_1^2-n_3^2)(n_2^2-n_3^2)}{\sqrt{n_1^2+n_3^2}\sqrt{n_2^2+n_3^2}\left(\sqrt{n_1^2+n_3^2}+\sqrt{n_2^2+n_3^2}\right)}, \tag{S6}$$

where $k_B = 1.38\times10^{-23}\,\mathrm{m^2\,kg\,s^{-2}\,K^{-1}}$ is the Boltzmann constant, T is the absolute temperature, $\nu_e \approx 3\times10^{15}\,\mathrm{s^{-1}}$ is the ionisation frequency of the materials, $h = 6.63\times10^{-34}\,\mathrm{m^2\,kg\,s^{-1}}$ is the Planck constant, and ϵ_i and n_i are the dielectric constant and refractive index of material i (where $i = 1, 2, 3$), respectively.

Supplementary Table S1. **Dielectric constants (ϵ) and refractive indices (n) of different materials.** These values are used to calculate Hamaker constants using Eq. S6.

Material	ϵ	n
Polydimethylsiloxane (PDMS)	2.7	1.4
Glass (Amorphous SiO_2)	3.82	1.46
Water	78.5	1.333

Table S1 summarizes the dielectric constants and refractive indices for the relevant materials in our experiments (36). Using the values in the table, we can calculate the Hamaker constant for a hydrophilic glass particle and a PDMS surface separated by a layer of water to be $A_{\mathrm{glass-water-pdms}} = 4.86\times10^{-21}$ J. Here, we used a temperature of $T = 293$ K. The corresponding Hamaker constant between a PDMS-coated hydrophobic particle and a PDMS surface separated by a water film is $A_{\mathrm{pdms-water-pdms}} = 3.87\times10^{-21}$ J. In both cases, the

Hamaker constant is positive, highlighting an attractive van der Waals interaction. Therefore, the presence of the water film can only be explained through other mechanisms, such as repulsive electric double layer forces between glass and PDMS in water.

S5. SUPPLEMENTARY TABLES AND FIGURES

Supplementary Table S2. **Simulation parameters.** Values of the parameters used in our numerical model to generate the phase diagram in Fig. 4A. To convert from simulation units to SI units, we first set the particle radius of 10 lattice units to be equivalent to 100 μm. This gives us the conversion factor for converting the length scale, $C_l = 10^{-5}$ m (*i.e.* 1 lattice units is equal to C_l). Then, we obtain the conversion factors for the mass and time, C_m, and C_t, respectively, by matching the surface tension (0.01) to the surface tension between air and water (72 mN/m) and the dynamic viscosity of the drop (0.0274) to that of water (1 mPa s). This procedure gives $C_m = 1.86 \times 10^{-14}$ kg and $C_t = 5.09 \times 10^{-8}$ s. All other quantities can be converted from simulation units to SI units from the conversion factors for length, mass, and time because the SI units of every other kinematic quantity are composed of metres, kilograms, and seconds. In experiments using water drops, particles are typically removed at the drop-air interface rather than by the bulk flow inside the drop, suggesting that the capillary force dominates the viscous force. Therefore, in our simulations, we chose the liquid-air surface tension, drop viscosity, and force applied to the drop, such the capillary force on the particle ($\sim \gamma R_p \sim 10^{-1}$) dominates the viscous force ($\sim \eta_d V R_p \sim 10^{-3}$, where V is the drop velocity in the simulations). To mimic the high viscosity contrast between water and air, we set the dynamic viscosity of the air to be 10 times smaller than the dynamic viscosity of the drop to ensure that the hydrodynamic force due to the flow inside the drop significantly exceeds any contribution from the flow of the surrounding air. The values for μ and F_p are chosen such that the friction force arising from the particle weight ($\mu F_p \sim 10^{-1}$) is comparable to the capillary force ($\gamma R_p \sim 10^{-1}$), to reproduce the outcome observed in our experiment with the hydrophobic particle. In addition to the values listed in this table, in Supplementary Figure S5, we varied the Bond number between 0.25 and 1.25. In Supplementary Figure S7, we varied μ and μ_r between 0 and 1.

Property	Symbol	Value (simulation units)	Value (SI units)
Time step	Δt	1	50.8 ns
Grid size	Δx	1	10 μm
Surface tension	γ	0.01	72 mN/m
Drop dynamic viscosity	η_d	0.0274	1 mPa s
Particle contact angle	θ_p	30°–150°	30°–150°
Surface contact angle	θ_s	30°–150°	30°–150°
Particle radius	R_p	10	100 μm
Normalized sliding friction of dry particle	$\mu F_p/(\gamma R_p)$	1.88	1.88
Normalized rolling friction of dry particle	$\mu_r F_p/(\gamma R_p)$	0.94	0.94
Drop (capillary bridge) radius in Fig. 4A	R_d	50	0.5 mm
Bond number applied to move drop in Fig. 4A	$f_d R_d^2/\gamma$	1.25	1.25

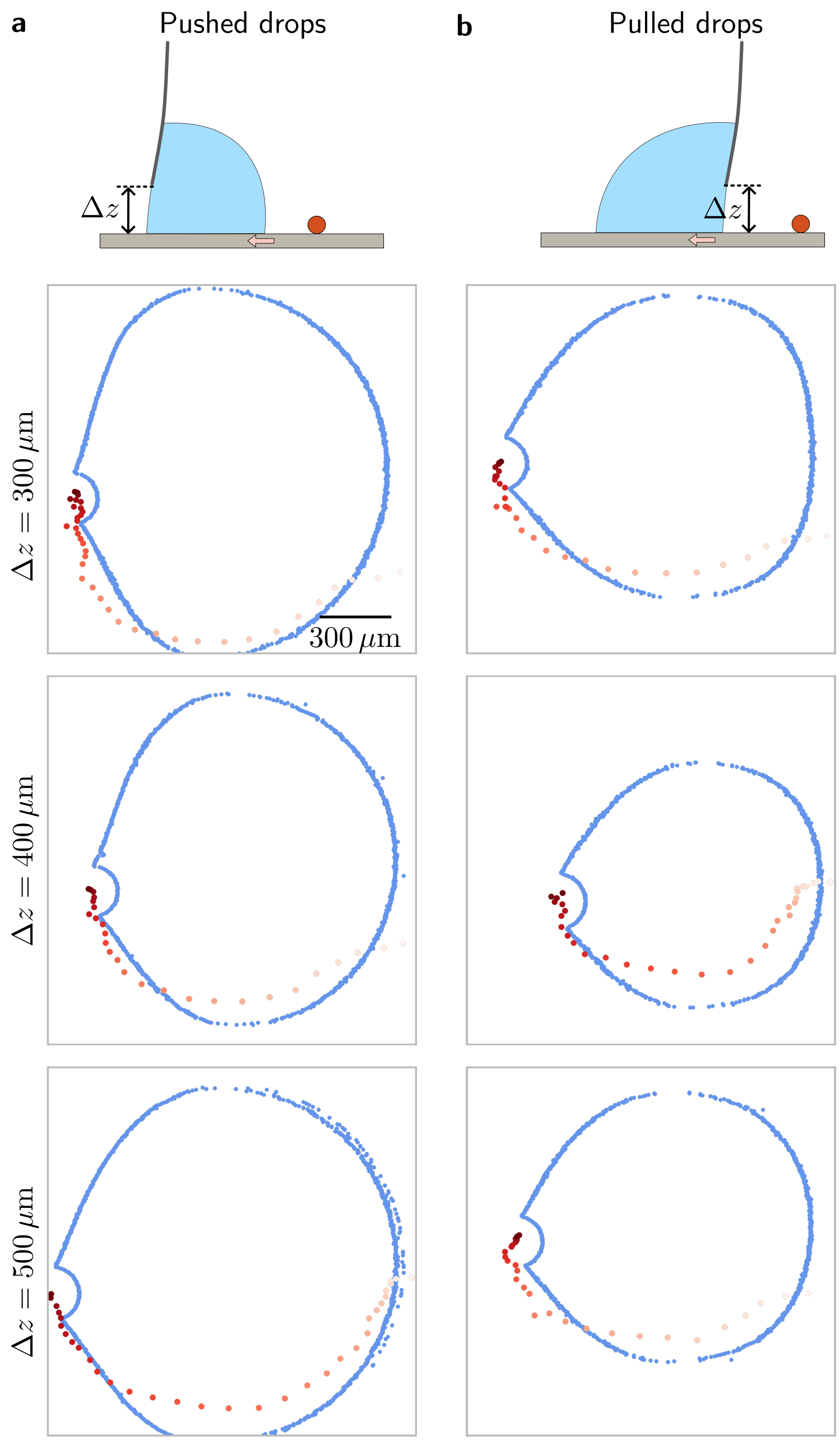

Supplementary Figure S1. **Effect of cantilever.** Varying the position of the cantilever to demonstrate that the cantilever does not affect the collision outcome. In these experiments, the particle diameter was 230 μm, the speed of the moving stage was 100 μm/s. In column (a), the cantilever was positioned at the rear side of the drop to push the drop, whereas in column (b), the cantilever was positioned at the front side of the drop to pull the drop. In both cases, the particle goes around the drop and remains attached at the rear side. The trajectory of the particle is shown by the red and orange scatter points, where darker colors correspond to later times. The contour shown for the drop corresponds to the final drop shape. Varying the spacing Δz between the substrate and the bottom edge of the cantilever did not influence the results.

Supplementary Table S3. **Contact angles and dynamic contact angle hysteresis between drop and surface.** Static contact angles, θ_s, and the corresponding dynamic advancing and receding contact angles, θ_a and θ_r, respectively, between the liquid and the solid surface. The simulation setup used here corresponds to a capillary bridge between two parallel solid surfaces, as shown in Supplementary Figure S4. The capillary bridge was initialized as a cylinder with radius 0.5 mm and height 0.56 mm. A horizontal force per unit volume of $6.92 \times 10^{-12}\,\mathrm{N/m^3}$ (5×10^{-6} in simulation units) was applied to move the capillary bridge to the right. Under this applied force, the capillary number of the capillary bridge, $\mathrm{Ca} = \eta_d V/\gamma$, was between 0.04 and 0.06, depending on θ_s. The phase diagram in Fig. 4 in the manuscript was also generated using the same applied force and capillary bridge dimensions. The static contact angle on the top surface was 90° and the static contact angle on the bottom surface was θ_s. There is no inherent static contact angle hysteresis in our model but dynamic contact angle hysteresis is still present. Here, the contact angles were extracted by fitting a tangent to the capillary bridge where the capillary bridge meets the bottom surface. In this set of simulations, no particle was included because the particle would interfere with the contact angle. For $\theta_s = 50°$, the receding angle is 0° since the drop stretches to form a film. Note that the values for the dynamic contact angle hysteresis ($\theta_a - \theta_r$) presented here only serve as a guide. They may differ when the drop interacts with the particle because the drop slows down during the interaction. Furthermore, the particle also deforms the drop and this may influence the contact angle locally.

θ_s (°)	θ_a (°)	θ_r (°)	$\theta_a - \theta_r$ (°)
50	65	0	65
70	83	55	28
90	101	78	23
110	119	98	21
130	139	116	23
150	158	132	26

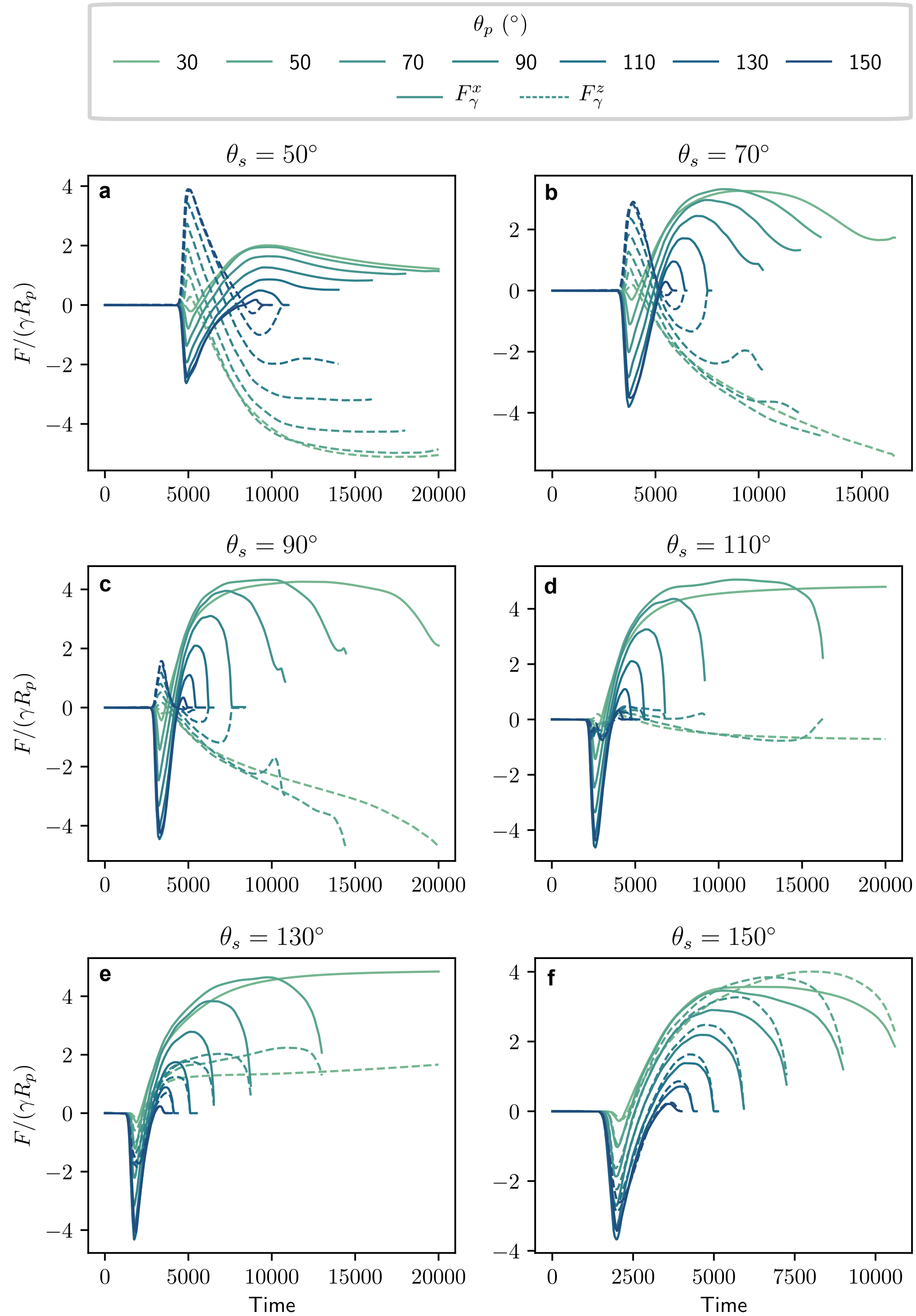

Supplementary Figure S2. **Capillary force acting on a particle as it detaches from a capillary bridge.** Solid lines are forces along x and dashed lines are forces along z. Each panel corresponds to a different contact angle θ_s between the liquid and the solid surface. Within each panel, the force curves for different particle contact angles θ_p are shown. The initial sharp jump (snap-in force) in the force curves corresponds to when the particle makes contact with the drop-air interface, and the interface quickly dewets the particle to reach equilibrium contact angle. This snap-in force is particularly large for high values of θ_p because the dewetting effect is strongest for more hydrophobic particles.

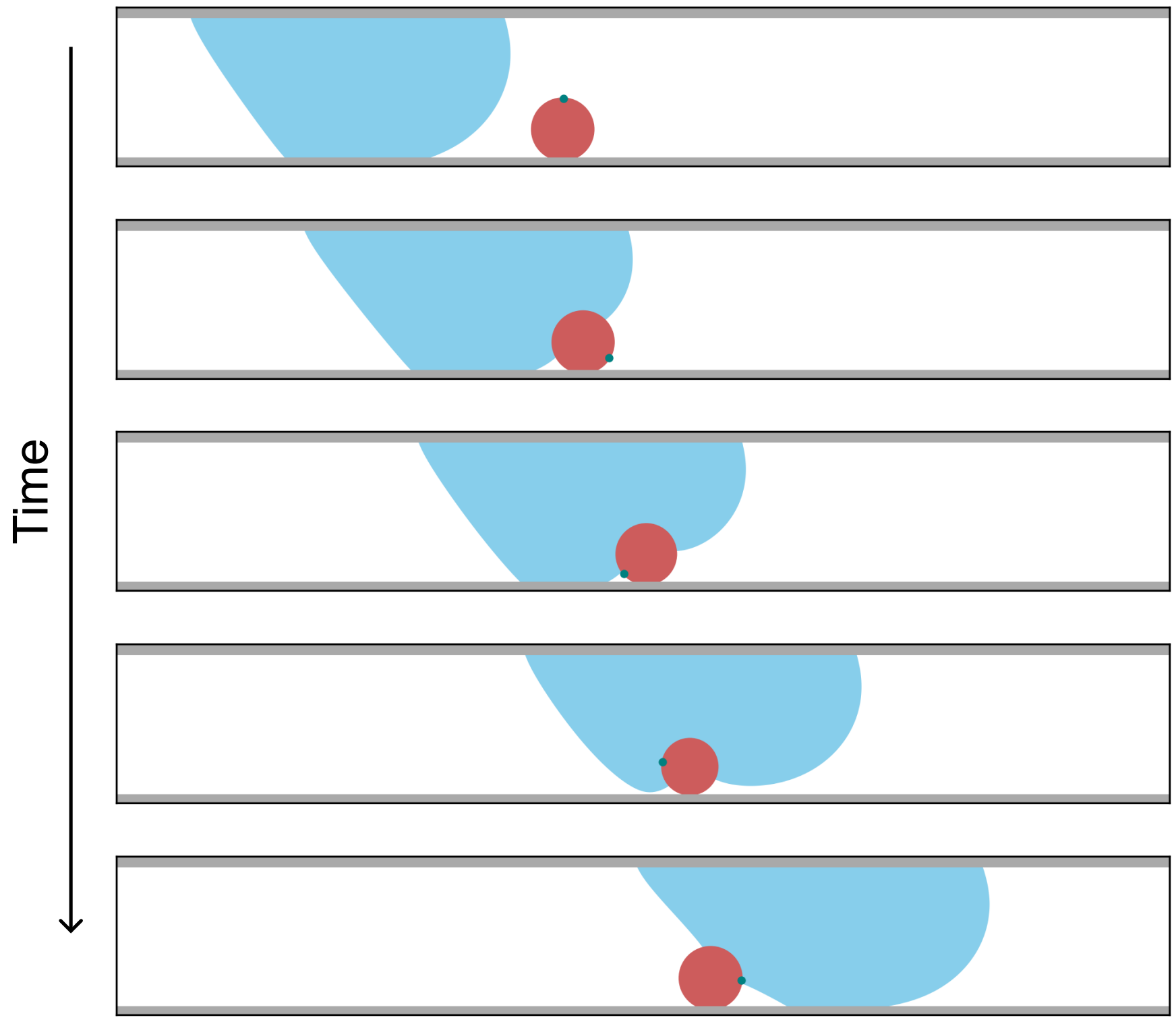

Supplementary Figure S3. **Particle travels under the drop and remains attached at the rear of the drop.** The simulation snapshots shown here correspond to the gray region of the phase diagram shown in Fig. 4A of the manuscript. The particle reaches the rear side of the drop by travelling under the drop. The green dot on the particle is added to track rotational motion.

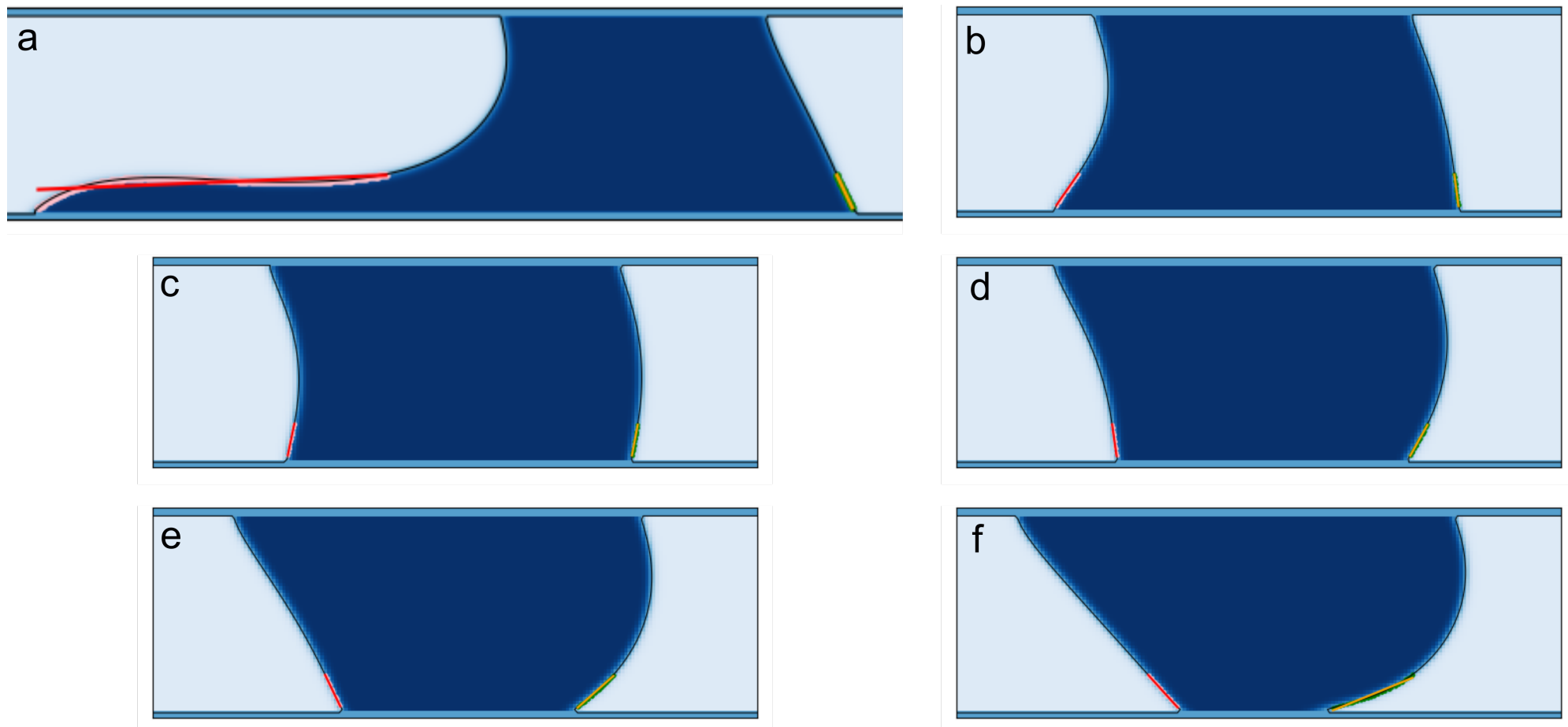

Supplementary Figure S4. **Measuring dynamic contact angle hysteresis for different static contact angles, θ_s.** Here, the drop is initialized as a cylinder between two parallel solid surfaces. The force applied on the drop per unit volume is 6.92×10^{-12} N/m^3 (5×10^{-6} in simulation units). The same configuration is used to generate the phase diagrams presented in Fig. 4A. On the top plate, $\theta_s = 90°$, whereas on the bottom plate, θ_s is varied from 50° to 150°. In (a)-(f), θ_s is 50°, 70°, 90°, 110°, 130°, and 150°, respectively. The contact angle is extracted by fitting a line (red line) to points (orange dots) on the liquid-air interface that lie within the first 10 lattice units above the bottom solid surface. In panel (a), the dynamic receding contact angle is taken to be 0° because the drop stretches to leave a film behind.

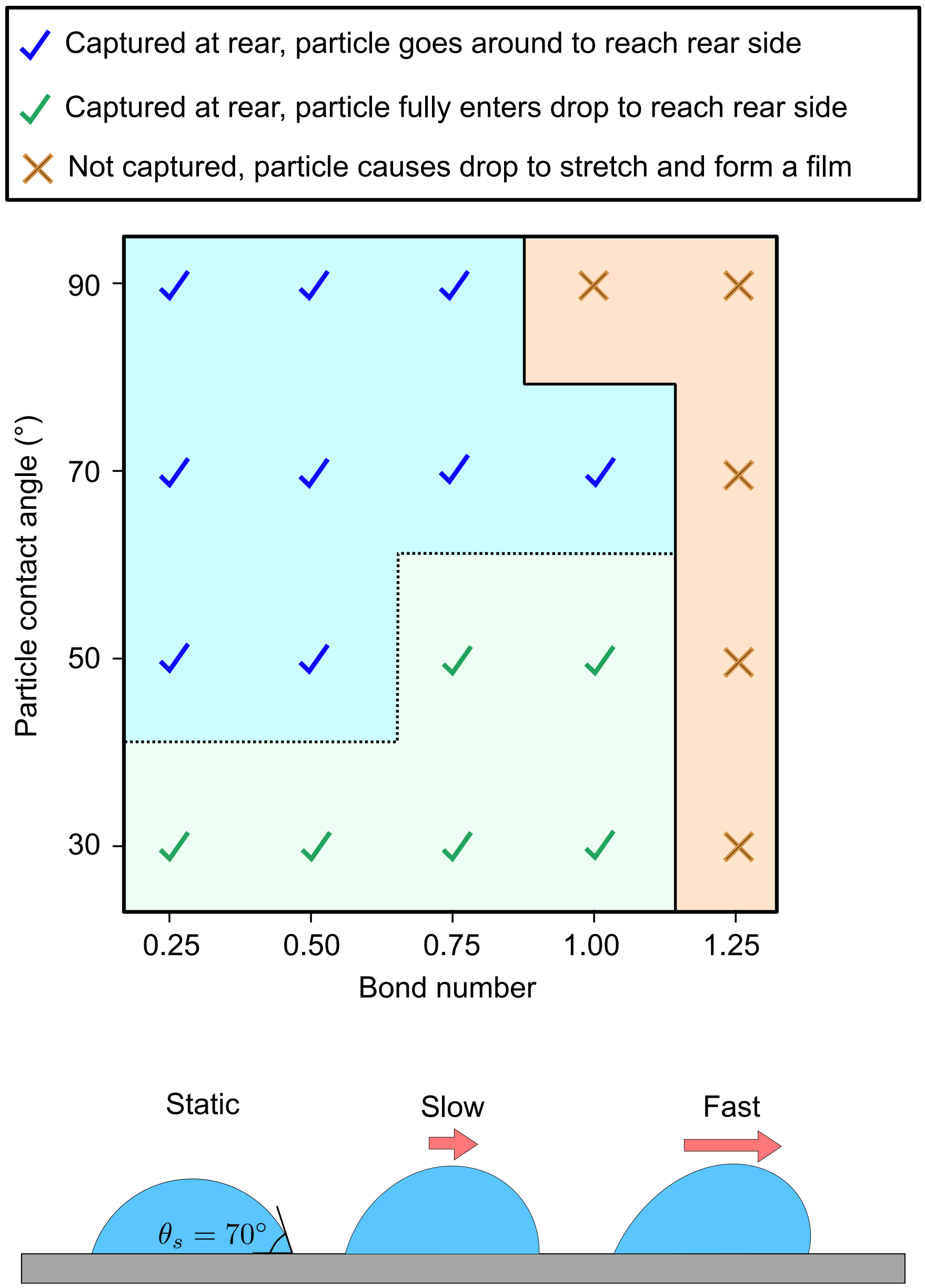

Supplementary Figure S5. **Variations in the observed outcomes as the force applied to move the drop increases.** Here, the Bond number (along the x axis) is defined as $Bo = f_d R_d^2/\gamma$, where f_d is the force applied to the drop per unit volume along x, $R_d = 0.5\,\text{mm}$ is the drop radius, and $\gamma = 72\,\text{mN/m}$ is the surface tension of the drop. The higher the Bond number, the higher the drop speed and thus the lower the dynamic receding contact angle. At high Bond numbers, the rear side of the drop forms a film, leaving the particle behind (orange region). Throughout this figure the static contact angle is $\theta_s = 70°$, and the drop speed varies by 1 order of magnitude between $\approx 0.2\,\text{m/s}$ to $\approx 2\,\text{m/s}$.

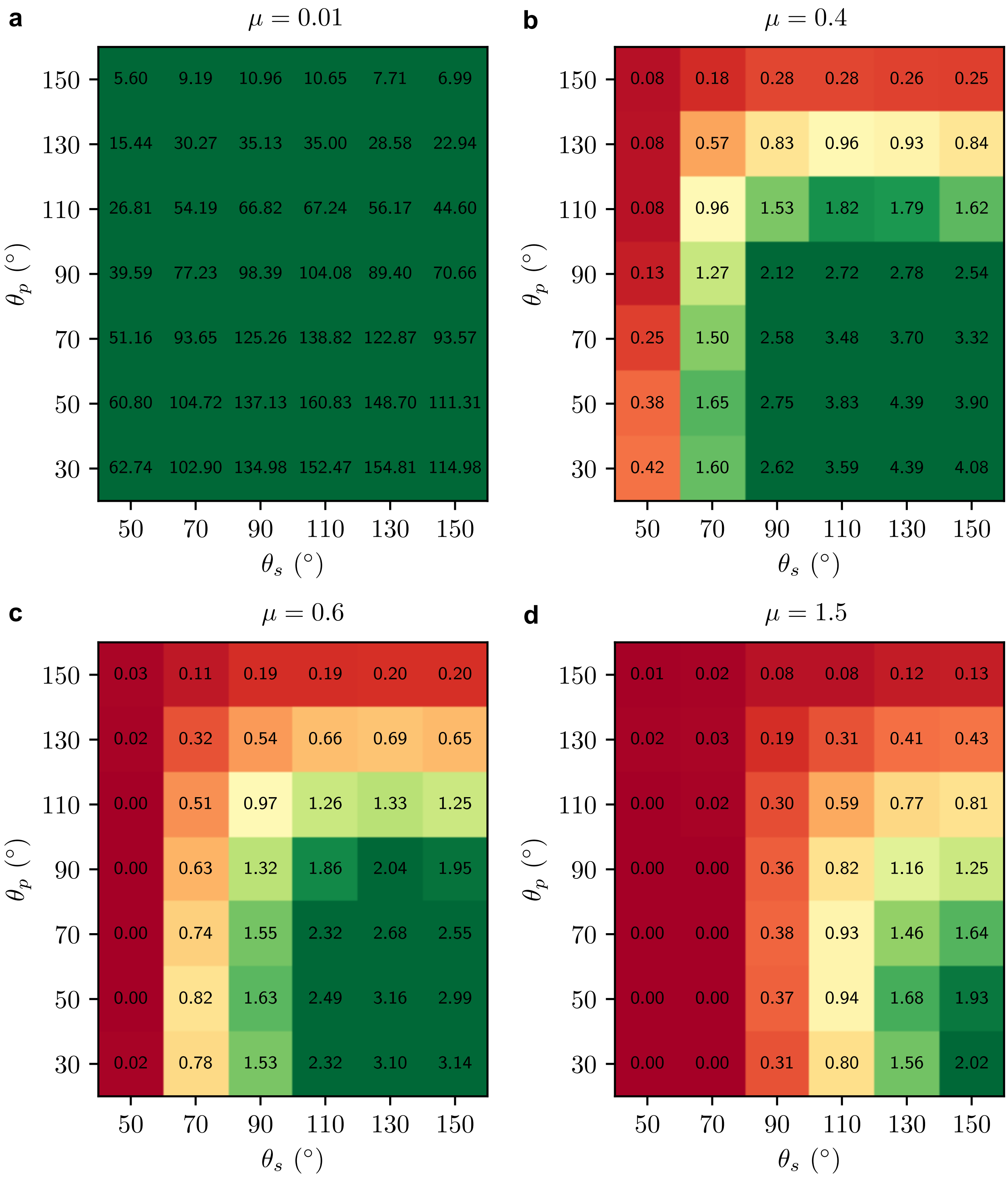

Supplementary Figure S6. **Variation of the capillary capture parameter, C, as a function of θ_s and θ_p for different friction coefficients, μ, between the particle and the surface.** The values written in the grid of the plot correspond to the value of C for different θ_s and θ_p. Successful particle capture corresponds to $C > 1$. The coefficients of friction chosen here are representative of the following material combinations: 0.01 corresponds to Teflon against Teflon, 0.4 corresponds to glass against glass, and 1.5 corresponds to aluminum against aluminum. Varying θ_p and θ_s corresponds to using different liquids to remove the particle. When $\mu = 0.01$, C is always > 1, predicting that the particle can always be captured. When μ increases, the region corresponding to $C > 1$ becomes narrower.

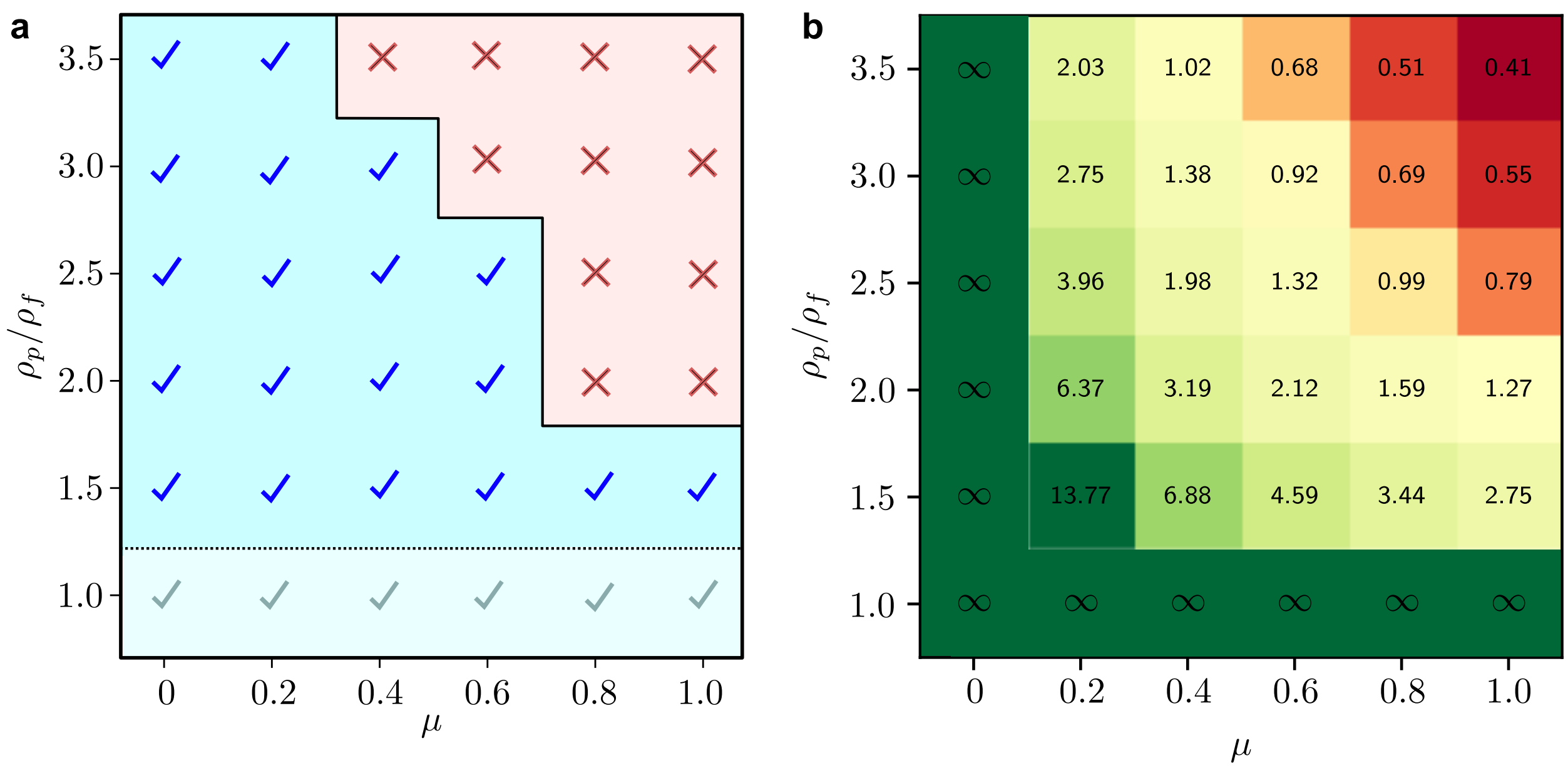

Supplementary Figure S7. **Variations in C as a function of the density ratio between the particle and the liquid, ρ_p/ρ_f and the coefficient of friction μ.** Here the coefficients of sliding and rolling friction are set to be equal, $\mu_r = \mu$. Varying ρ_p/ρ_f changes the vertical force F_p acting on the particle, and thus also the friction force. Throughout the rest of the paper, ρ_p/ρ_f was set to 2.5. The numerically observed outcomes obtained when independently varying the friction coefficient and ρ_p/ρ_f are shown in (a). When $\rho_p/\rho_f = 1$ (bottommost row), the particle is lifted off the surface due to buoyancy. Panel (b) shows the values of C as predicted by our semi-analytical expression (Eq. 5 in the manuscript). The values written in the grid of the plot correspond to the value of the capillary capture parameter C.
